# Hendra Virus and Horse Owners – Risk Perception and Management

**DOI:** 10.1371/journal.pone.0080897

**Published:** 2013-11-15

**Authors:** Nina Kung, Amanda McLaughlin, Melanie Taylor, Barbara Moloney, Therese Wright, Hume Field

**Affiliations:** 1 Queensland Centre for Emerging Infectious Diseases, Biosecurity Queensland, Department of Agriculture, Fisheries and Forestry, Brisbane, Queensland, Australia; 2 Centre for Health Research, School of Medicine, University of Western Sydney, Sydney, New South Wales, Australia; 3 Biosecurity New South Wales, Department of Primary Industries, Orange, New South Wales, Australia; 4 EcoHealth Alliance, New York, New York, United States of America; 5 Animal Biosecurity & Welfare Program, Biosecurity Queensland, Department of Agriculture, Fisheries and Forestry, Brisbane, Queensland, Australia; University of Hong Kong, Hong Kong

## Abstract

Hendra virus is a highly pathogenic novel paramyxovirus causing sporadic fatal infection in horses and humans in Australia. Species of fruit-bats (genus *Pteropus*), commonly known as flying-foxes, are the natural host of the virus. We undertook a survey of horse owners in the states of Queensland and New South Wales, Australia to assess the level of adoption of recommended risk management strategies and to identify impediments to adoption. Survey questionnaires were completed by 1431 respondents from the target states, and from a spectrum of industry sectors. Hendra virus knowledge varied with sector, but was generally limited, with only 13% of respondents rating their level of knowledge as high or very high. The majority of respondents (63%) had seen their state’s Hendra virus information for horse owners, and a similar proportion found the information useful. Fifty-six percent of respondents thought it moderately, very or extremely likely that a Hendra virus case could occur in their area, yet only 37% said they would consider Hendra virus if their horse was sick. Only 13% of respondents stabled their horses overnight, although another 24% said it would be easy or very easy to do so, but hadn’t done so. Only 13% and 15% of respondents respectively had horse feed bins and water points under solid cover. Responses varied significantly with state, likely reflecting different Hendra virus history. The survey identified inconsistent awareness and/or adoption of available knowledge, confusion in relation to Hendra virus risk perception, with both over-and under-estimation of true risk, and lag in the uptake of recommended risk minimisation strategies, even when these were readily implementable. However, we also identified frustration and potential alienation by horse owners who found the recommended strategies impractical, onerous and prohibitively expensive. The insights gained from this survey have broader application to other complex risk-management scenarios.

## Introduction

Hendra virus was first described in 1994 in Australia when it caused disease and death in horses and close-contact humans [1,2]. The virus is highly pathogenic and effective treatment does not currently exist. While infectivity, and the incidence of disease is low, the case fatality rate is high, with around 80% of equine cases and 60% of human cases having a fatal outcome [3,4]. Species of fruit-bats (sub-order *Megachiroptera*, genus *Pteropus*), commonly known as flying-foxes, are the natural host of the virus, and evidence of infection has been found in all four species (*Pteropus alecto*, *P. conspicillatus*, *P. poliocephalus*, *P. scapulatus*) occurring on mainland Australia [5]. Flying-foxes are nomadic and colonial species [6], and are periodically or continuously present in rural, peri-urban and urban environments across their geographic range. Virus has been detected in the urine, faeces, saliva and birthing fluids of experimentally infected flying-foxes [7–9], and in the urine, uterine fluid and foetal tissue of naturally infected free-living flying-foxes [10,11]. Field et al, 2011 [10,11] reported variable virus excretion in urine, with prevalence in pooled urine samples collected under roosting flying-foxes ranging from 3-33% in the one-in-four sampling events that yielded positive results. Subsequent studies have detected excretion spikes as high as 60% on rare occasions (Field et al unpublished data). Transmission to horses is believed to follow oro-nasal contact with the body fluids of infected flying-foxes [1,5,7], plausibly while grazing. Prior to 2011, thirteen of the 14 known incidents had occurred in the state of Queensland (QLD), with only single case occurring in the adjacent state of New South Wales (NSW). In 2011, an unprecedented 18 incidents occurred, ten in QLD and eight in NSW [12]. All human cases are attributed to direct contact with the body fluids of infected horses [4,13,14]. Strategies for managing exposure risk in horses focus on minimising potential equine contact with flying-fox body fluids; strategies for managing human exposure risk focus on avoiding direct and unprotected contact with sick horses [15,16]. The nomadic (and nocturnal) life history traits of flying-foxes preclude effective management of their movements, so risk minimisation strategies target the horse environment at an individual property level, and include recommendations such as covering feed bins and water troughs, and excluding horse access to pasture beneath flowering or fruiting trees in which flying-foxes are feeding [16]. Thus, the onus falls on the individual horse owner to modify facilities, adapt husbandry practices and restrict horse movements. While a vaccine for horses has recently been marketed in Australia [17], uptake has been limited. Adoption of risk minimisation strategies requires knowledge, consideration, decision and action by the horse owner. We undertook a survey of horse owners to understand their knowledge, attitudes, opinions and actions in relation to Hendra virus. The objective was to assess the level of adoption of the recommended strategies and identify impediments to adoption. 

## Methods

### Study population

Our target study population was horse owners in the (to date) Hendra virus-affected states of Queensland and New South Wales, and our promotion targeted these two states. While we accepted responses from all Australian states (given the national profile of Hendra virus), our analysis focused on responses from Queensland and New South Wales.

### Questionnaire

We posed 46 questions within 6 sections, capturing respondent demographic information, property and management details, property vegetation and flying-fox profiles, perceived Hendra virus risk, awareness of Hendra virus risk management recommendations, and attitudes and risk management actions. Questions were typically closed, though there were also comment boxes for most of the questions for respondents to make additional comment. The questionnaire was trialled across a cross-section of horse owner profiles and refined accordingly prior to the start of the survey. 

### Survey delivery

We presented the survey in a web-based on-line format using the SurveyMonkey^®^ platform, though advertised the availability of paper copies to potential respondents who did not have internet access. The survey commenced on 9 January 2012 and continued for 12 weeks until 31 March 2012. It was promoted by the Queensland and New South Wales state governments via conventional media release and social media in weeks 1 and 10, and via several radio and print media interviews in the intervening period. In addition, a number of horse industry groups posted the survey URL on their official websites. 

### Analysis

We used the SurveyMonkey^®^ program to collect, store and manipulate the data, and Microsoft^®^ Office Excel program to derive descriptive summary statistics. P values of the chi square statistic were used to examine statistical significance at the 95% confidence level. We analysed the responses to all questions, but have focused the analysis presented here on the fundamental issues of horse owner knowledge, horse owner risk perception, and horse owner risk mitigation actions. Response percentages were rounded to the nearest whole number. The eleven industry sector categories in Question 1 were collapsed into 7 categories for analysis as follows: ‘recreational’, ‘equestrian’, ‘thoroughbred and harness racing’, ‘breeding and agistment’, ‘commercial’, ‘veterinary and para-veterinary’ and ‘farm’. 

### Ethics

The survey and questionnaire were reviewed by the Human Research Ethics Committee of Queensland Health, and approved as a low/negligible impact process (HREC Reference number: HREC/11/QHC/56). Respondents were required to indicate informed consent by clicking a button to access the on-line questionnaire. 

## Results

**Figure 1:**
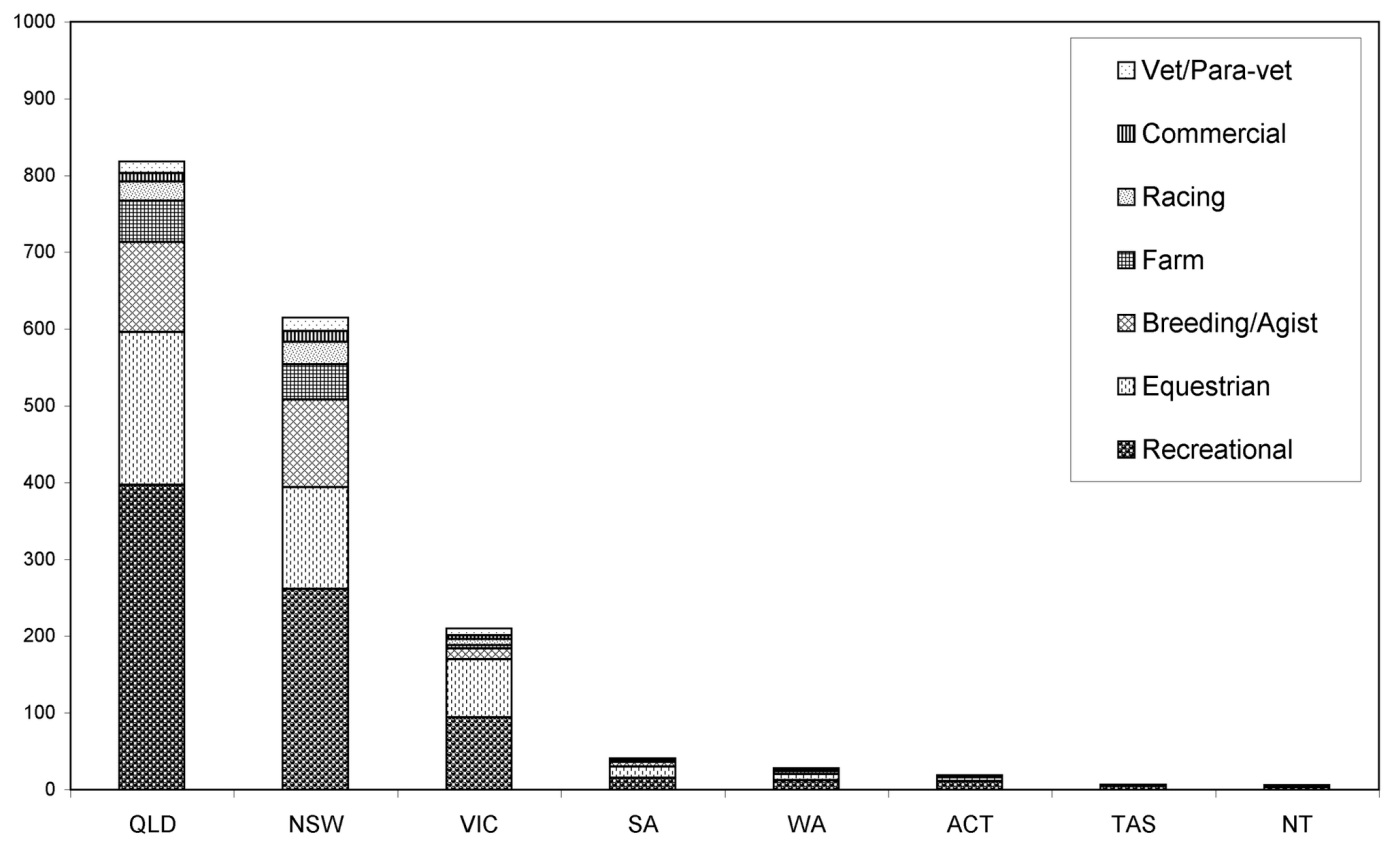
The number of respondents completing the questionnaire from each Australian state or territory with industry sector composition. (QLD = Queensland, NSW = New South Wales, VIC = Victoria, SA = South Australia, WA = Western Australia, ACT = Australian Capital Territory, TAS = Tasmania, and NT = Northern Territory.)

**Figure 2:**
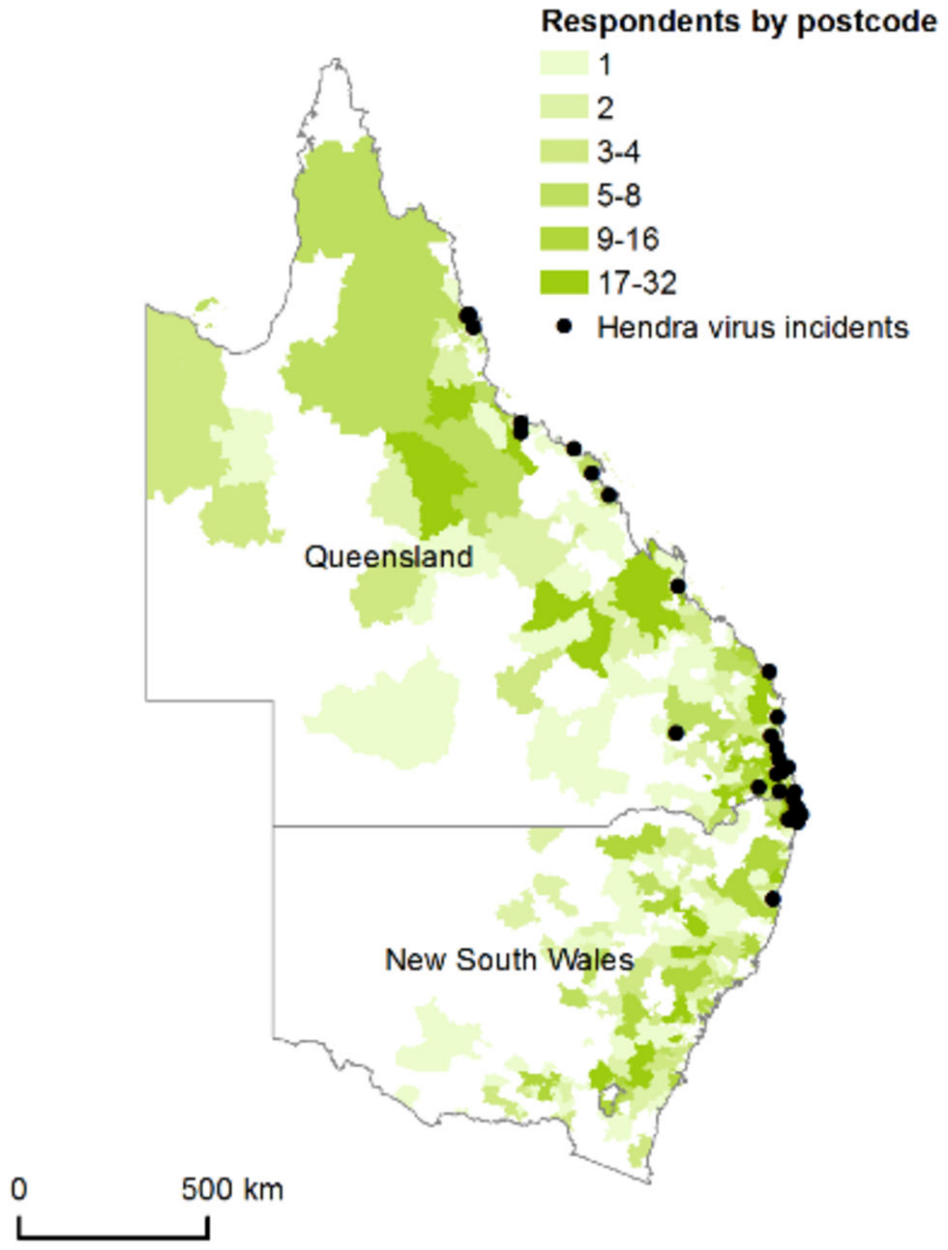
Map of eastern Australia illustrating the spatial distribution of the 1431 respondents from the target study population of Queensland and New South Wales, and indicating reported Hendra virus case locations.

A total of 1850 respondents accessed the survey, with 1744 completing the questionnaire (Figure 1). We analysed the responses from 1431 respondents from the target study population in Queensland and New South Wales. Number of respondents from different postcodes in Queensland and New South Wales were mapped in Figure 2 together with the locations of reported Hendra virus case properties (horses and human). The demographic characteristics of these respondents are presented in Table 1. Ninety-nine percent of respondents completed the questionnaire on-line. Summary statistics for selected questions that address the fundamental survey focus are presented below.

**Table 1:** Demographic characteristics of the 1431 respondents from the target study population in Queensland and New South Wales.

Variable	Category	Number (%) of Respondents
**Education**		
	Year 10 or less	215 (15)
	Year 12 or HSC	272 (19)
	Certificate or Diploma	452 (31)
	Bachelor's Degree	296 (21)
	Postgraduate Qualification	196 (14)
**Age**		
	Under 16	24 (2)
	16-24	126 (9)
	25-34	198 (14)
	35-44	337 (23)
	45-54	444 (31)
	55-64	236 (16)
	>65	66 (5)
**Gender**		
	Female	1210 (85)
	Male	221 (15)
**Income Derived From Horses**		
	Yes	245 (17)
	No	1186 (83)
**Industry Sector**		
	Breeding and Agistment	231 (16)
	Commercial	25 (2)
	Equestrian	332 (23)
	Farming	100 (7)
	Recreational	656 (46)
	Racing	54 (4)
	Veterinary and Para-veterinary	33 (2)
**State**	Queensland	814 (57)
	New South Wales	617 (43)
**Case postcode**	Queensland	167 (12)
	New South Wales	46 (3)

### Hendra virus knowledge

Self-rated horse owner knowledge of Hendra virus was generally limited, with only 13% (n = 178) of respondents rating their level of knowledge as high or very high; a further 53% (n = 697) rated their level of knowledge as moderate. The level of knowledge varied significantly with state (Table 2) and with industry sector. Regarding the latter, the proportion of respondents who indicated a moderate, high or very high level of knowledge varied from 53% (n = 28) (racing sector) to 87% (n = 26) (veterinary/para-veterinary sector) (χ^2^ = 14.2, *p* = 0.028). Knowledge did not vary with education level. Seven percent of respondents left this question blank.

**Table 2:** Hendra virus knowledge^1^, risk perception and risk management among survey respondents.

	**QLD**		**NSW**		
	Total # responses^2^	# (%) of respondents who indicated this response	Total # responses^2^	# (%) of respondents who indicated this response	Chi-square value^3^,*p* value
Moderate, high or very high knowledge of HeV	755	581 (77)	572	294 (51)	94.6, <0.0001
Limited or no knowledge of HeV	755	174 (23)	572	278 (49)	94.6, <0.0001
Have seen state’s HeV information pack for horse owners	750	577 (77)	566	255 (45)	141.0, <0.0001
Information in the pack was useful	750	515 (69)	566	251 (44)	78.4, <0.0001
Water point near or under trees	791	127 (16)	605	159 (26)	22.0, <0.0001
Water point under solid cover	791	165 (21)	605	46 (8)	47.0, <0.0001
Spoke with private veterinarian about HeV	755	409 (54)	572	211 (37)	39.1, <0.0001
Very likely or extremely likely that an HeV case could occur in my area	755	250 (33)	572	113 (20)	29.2, <0.0001
Very or extremely concerned that your horse could become infected if there was a local case	755	399 (53)	572	394 (69)	34.8, <0.0001
Very or extremely concerned that you could become infected if there was a local case	755	306 (41)	572	330 (58)	38.4, <0.0001
Would consider HeV if horse was unwell	755	350 (46)	572	144 (25)	62.5, <0.0001

^1^ Hendra virus knowledge was self-assessed by respondents.

^2^ Total number of responses excludes those respondents who left a question blank.

^3^ Degrees of freedom equal one for all categories.

Nearly two-thirds of all respondents had seen a Hendra virus information pack for horse owners, with a significantly greater proportion of QLD respondents having done so. Similarly, a significantly greater proportion of QLD respondents felt the information was useful to them in reducing Hendra virus infection (Table 2). Industry sector perspective on usefulness varied non-significantly, with 50% (farm sector, n = 42) to 80% (veterinary/para-veterinary sector, n = 24) finding the information useful (χ^2^ = 8.81, *p* = 0.185), and 0% (veterinary/para-veterinary sector, n = 0) to 10% (agistment/breeding sector, n = 22) not finding the information useful (χ^2^ = 10.2, *p* = 0.116). Responses did not vary significantly with education level. Ten percent of respondents gave additional written comments, with the most frequent comments being that the information was impractical and not in layman’s terms, the risk-mitigation strategies were expensive to implement, and that more focus should be on flying-fox control. These respondents were predominantly from the recreational (50%, n = 65), equestrian (20%, n = 26), and agistment/breeding (18%, n = 23) sectors. Eight percent of respondents left this question blank.

Awareness of the main clinical signs associated with Hendra virus infection in horses was generally good, with 83% (n = 1067) of respondents indicating respiratory signs, 82% (n = 1053) indicating neurological signs, 70% (n = 905) indicating fever, 53% (n = 683) indicating colic-like symptoms, and 52% (n = 673) indicating a high heart rate. Awareness was similar in both QLD and NSW. Ten percent of respondents left this question blank.

Nearly half of all respondents had spoken with their private veterinarian about how to limit the risk of Hendra virus infection. QLD respondents were significantly more likely to have done so (Table 2), and racing sector respondents were almost significantly less likely to have done so (χ^2^ = 3.61, *p* = 0.057). A minority of respondents (14%, n = 191) had spoken with a government veterinarian or livestock inspector (QLD 18%, n = 132; NSW 10%, n = 59). 

State government sources and horse interest group sources ranked first (37%, n = 463) and second (27%, n = 345) respectively as the main source of Hendra virus information overall. This order was the same in QLD and NSW, however the rankings for 3^rd^ and 4^th^ varied between states, with QLD respondents identifying veterinary advice (11%, n = 80) before television (6%, n = 40), in contrast to NSW respondents 10% (n = 53) and 15% (n = 79) respectively. Only 33% (n = 417) of all respondents indicated veterinary advice in their top three information sources. 

### Risk perception

Just over a quarter of all respondents thought it was very likely or extremely likely that a Hendra virus case could occur in their area, with a significantly greater proportion of QLD respondents thinking so (Table 2). A further 29% of all respondents (n = 386) believed the chance of a Hendra virus case occurring locally was moderately likely, while 38% (n = 500) believed such a scenario was a little likely or not at all likely (QLD 29%, n = 222, NSW 49%, n = 278). The proportion of respondents who thought it very likely or extremely likely that a Hendra virus case could occur in their area also varied non-significantly across industry sectors (χ^2^ = 6.83, *p* = 0.336), from 20% (farm sector, n = 17) to 41% (commercial sector, n = 9). Overall, respondents from NSW postcodes where a Hendra virus case had previously occurred had the highest perceived risk, with 85% (n = 39) believing it was very likely or extremely likely that a case could occur in their area. In contrast, 30% of QLD respondents (n = 50) from such postcodes believed a case was very likely or extremely likely in their area (χ^2^ = 44.6, *p* < 0.0001). 

Nearly two thirds of all respondents were very concerned or extremely concerned that their horse could become infected if there was a local Hendra virus case, with a significantly greater proportion of NSW respondents indicating so. Similarly, almost half of all respondents were very concerned or extremely concerned that they personally might be infected if there was a local equine case, again with a significantly greater proportion of NSW respondents indicating so (Table 2). 

Combining both states’ responses, respondents from case postcodes were significantly less likely than respondents from non-case postcodes to be very or extremely concerned that they (χ^2^ = 7.36, *p* = 0.007) or their horse (χ^2^ = 5.44, *p* = 0.020) could become infected if there was a local case.

Thirty-six percent of respondents (n = 518) observed their horse sometimes or often eats leaves of trees or shrubs in the paddock. Three percent of respondents (n = 40) had seen flying-foxes come to the horse feed or water points sometimes, frequently or every night. 

Finally, just over a third of all respondents indicated they would consider Hendra virus if their horse seemed generally unwell, with QLD respondents proportionately more likely than NSW respondents to do so (Table 2). 

### Risk mitigation actions

Horses were routinely stabled overnight by 13% (n = 179) of all respondents. Of the remainder, 24% indicated it would be easy or very easy to do, and 65% indicated it would be difficult, very difficult or impossible. Responses did not vary markedly between states or among industry sectors.

Ninety-five percent of all respondents provided some supplementary feeding. Thirteen percent (n = 183) and 15% (n = 211) of respondents respectively had horse feed bins and water points under solid cover, and 3% (n = 43) and 20% (n = 286) respectively had feed and water points near or under trees. Fifty-one percent of respondents (n = 727) said it would be easy or very easy to move feed and water points away from trees. Fifteen percent (n = 221) said it would be difficult, very difficult or impossible to do so. However, 67% (n = 956) of respondents indicated it would be difficult, very difficult or impossible to move feed and water points under solid cover. The latter included the 47% of respondents (n = 672) who have a dam or stream as either the sole water source, or one of several water sources. The most frequent comments about moving or covering feed and water points were that it was impractical, or that the cost was prohibitive. Water point location varied significantly between states (Table 2).

When asked how easy it would be to remove their horse from paddocks when trees were fruiting or flowering, 29% (n = 421) said it would be easy or very easy, but 51% (n = 733) said it would be difficult, very difficult or impossible. Responses did not vary markedly between states. The most frequent comments about excluding horses from fruiting or flowering trees related to neighbouring property trees along the fence-line, cost, and property layout (e.g. the impracticality of excluding horses from creek-lines).

A Hendra virus vaccine for horses would probably or definitely be used by 80% of all respondents (n = 1303). In contrast, 6% (n = 80) said they would probably not or definitely not vaccinate. Responses did not vary significantly across state or industry sector. The most frequent comments for non-vaccination related to cost, safety and efficacy, and risk perception.

## Discussion

Hendra virus infection is topical and contentious with horse owners, veterinarians and para-veterinary professions in Australia because of its cryptic bat origin, its typically fatal outcome, and its zoonotic potential [18,19]. Infection in horses has a complex causality, and it is evident that horse management practices can mitigate exposure risk [20]. Animal health authorities have long provided risk management advice to horse owners [15,16], however the increased number of cases and the expanded geographic occurrence of cases in recent years make it timely to review the level of adoption of the recommended strategies, identify impediments to adoption, and to canvass additional strategies. All confirmed or possible equine Hendra virus cases have occurred in the eastern states of Queensland and New South Wales [15], thus horse owners in these states were the target of our survey. We have focused in reporting the responses in the context of three over-arching categories: Hendra virus knowledge, risk perception, and risk mitigation actions which have provided more insight information. 

### Respondent demographic characteristics

With no sampling frame available, we undertook wide and diverse promotion of the survey to reach horse owners in all sectors of the horse industry. While the relative proportion of industry sectors is not definitively known, the racing sector is likely under-represented in the survey based on anecdotal evidence, and thus the survey findings should be interpreted with care in relation to this sector. This sector was challenging to engage, surprisingly, given that the first described Hendra virus outbreak was in a racing stable. 

The skewed gender response suggests a response bias, with female respondents potentially over-represented, however anecdotal evidence (e.g. club memberships) indicates that horse ownership in eastern Australia is positively skewed towards females. However, to ascertain the significance of a potential gender response bias, we analysed key responses by gender, and found no significant difference in Hendra virus knowledge or risk perception, with the singular exception of a heightened ‘extreme risk’ perception by female respondents that a case could occur in their area (data not shown).

### Hendra virus knowledge

Disconcertingly, a higher proportion of respondents indicated little or no knowledge of Hendra virus than indicated a high or very high knowledge. This response intuitively suggests limited success of the current communication strategy based on state government and industry-provided information via internet, printed material and live forums. However, the response and this interpretation are at odds with the high proportion of horse owners who have seen their state’s Hendra virus guidelines/information pack for horse owners (nearly two thirds), and who regarded the information as useful in minimising Hendra virus risk. A plausible interpretation of this anomaly is that horse owners are accessing the information, but are not actually being informed, perhaps because of the volume of material, the complexity of the epidemiology of Hendra virus infection, or the way the information is presented. 

Alternatively, it may suggest that horse owner focus is narrow, and that they primarily seek details that are directly relevant to their specific situation. This latter interpretation is consistent with the generally high level of knowledge of the most common clinical signs associated with Hendra virus infection in horses. The lower level of knowledge and information access in NSW likely reflects the more recent profile of Hendra virus in that state, subsequent to the 2011 cluster involving 10 cases; prior to this, only a single case had occurred in NSW, in 2006. Similarly, the higher response in NSW to television as a source of information over veterinarians likely reflects the novelty of Hendra virus in that state. However, it would also likely contribute to the lower level of horse owner knowledge in NSW, given the typically superficial coverage of Hendra virus incidents by television. It is concerning, nonetheless, that only one third of all respondents included veterinary advice in their top three information sources, suggesting a missed opportunity for private veterinary practitioners to fulfil client needs. The varying level of Hendra virus knowledge across industry sectors in part reflects the professional need for veterinarians to have a high level of knowledge of infectious diseases; a lower level of knowledge by some sectors may reflect the pressure of competing knowledge demands on individuals in broad-based sectors such as farming. The challenge is to package and convey key information accordingly. 

### Risk perception

In formal risk analysis terms, risk is defined as having two components: likelihood and consequence. One of the challenges with Hendra virus risk perception is getting this balance right. There have been 80 confirmed or possible equine cases and 7 human cases recorded in the 18 years since the first detected case in August 1994 [21,22], so the true likelihood of infection in both horses and humans is low given the estimated number of horses (>500,000) and people (>1,000,000 assuming two potential human risk exposures per case horse) theoretically at risk in QLD and NSW annually. However, while the likelihood of infection is low, the consequences of infection can be dire given the high case fatality rate. Media reporting generated by the latter tends to skew horse owner perception of risk, so it is not surprising that respondents from postcodes with a previous Hendra virus case have an elevated perception of likelihood of a case in their area. Another challenging aspect of communicating Hendra virus risk is the frequent confusion by horse owners about the infectivity of a case horse. Hendra virus is not highly infectious, and effective transmission appears to require direct contact with infectious body secretions or excretions, yet the consequences of infection (in contrast to the likelihood of infection) mean that suspect or case horses (and properties) need to be handled with a high level of biosecurity. The situation is compounded by a tendency to (wrongly) equate the high pathogenicity of Hendra virus (that is the demonstrated virus’s ability to cause serious disease) with high infectivity (that is, the ability to transmit readily from horse to horse, or from horse to human). However, the tendency to over-estimate the likelihood of a case by some respondents is contrasted by the tendency to under-estimate the likelihood by others, with approximately one in three respondents feeling there was little or no likelihood of a case in their area. There was also variation in risk perception across industry sectors, with respondents in the commercial sector perceiving a greater likelihood of a case in their area. This elevated risk perception may reflect anxiety regarding anticipated negative economic consequences in the event there was a case. 

Clearly, the subject of risk communication and perception requires additional and targeted effort. This situation is underlined by the alarming finding that only about one third of respondents would consider Hendra virus if their horse was unwell, and paradoxically by almost half the respondents who were very concerned or extremely concerned that they personally might be infected if there was a local equine case. 

### Risk mitigation actions

Several Hendra virus infection risk mitigation strategies for horses have been promoted by the relevant state government departments, including removing feed and water points from under trees, placing feed and water points under roofed structures, night stabling, and excluding horses from access to flowering and fruiting trees in the paddock. Smith et al [23] found that almost 100% of urine and food debris from foraging flying-foxes fell under the tree canopy, suggesting that moving feed bins and water points from underneath trees was an effective way of reducing horse contact with potentially infectious materials. This survey found that 3% and 20% of respondents respectively had feed and water points near or under trees, and that half said it would be easy or very easy to move feed and water points away from trees. Some respondents commented that they placed water troughs under trees to reduce evaporation, especially when trees were the only shade in the paddock. While 13% and 15% of respondents respectively had placed feed bins and water points under a roofed structure, two thirds indicated it would be difficult, very difficult or impossible to move feed and water containers under solid cover. The difference in water point management between states may reflect the lower Hendra virus profile in NSW prior to 2011.

Night stabling is an effective risk management strategy in that it removes the horse from direct contact with potentially infectious materials from nocturnally foraging flying-foxes. While it doesn’t preclude contact with environmental contamination the following day, experimental studies indicate that viral load is rapidly reduced by temperature and desiccation [24], suggesting that the window of opportunity for contact with infectious materials is shorter in the day and longer at night. While 13% of respondents routinely stabled their horse overnight, a further 24% said it would be easy or very easy to do. In contrast, two thirds of respondents said it was impractical or financially prohibitive to stable their horses overnight. Some respondents commented that they put their horse(s) into a small night paddock thinking it would reduce Hendra virus exposure risk. Paradoxically, such paddocks are typically close to the house and associated garden/fruit trees which may attract flying-foxes. Finally, nearly one third of respondents said it would be easy or very easy to remove their horse from paddocks when trees were fruiting or flowering. 

Thus, effective risk mitigation actions can be readily implemented by a substantial proportion of respondents, with removal of feed bins and water points from near or underneath trees being the easiest. These actions have been proposed for several years, particularly in the Queensland Hendra virus Information for Horse Owners on-line brochure [16], yet it would seem that either a substantial number of horse owners remain unaware of them, or are aware of them but have not implemented them. These drivers require better understanding, and potentially more efficient or targeted communication. However, notwithstanding this unrealised potential for feasible risk reduction, many respondents commented that the recommended risk management strategies were impractical in their circumstances, were onerous, or were prohibitively expensive. This is illustrated by the farm sector response regarding usefulness of the information to horse owners, with only 50% responding positively. Some respondents expressed frustration that what they perceived as more simple and direct solutions (e.g. removing trees or controlling flying-foxes) were impeded by government regulations. Such comments indicate the potential for the disillusionment and alienation of some horse owners, and warrant contemplation. 

An effective vaccine for Hendra virus has recently become available, promising protection from infection for vaccinated horses. When asked if they would probably or definitely use the vaccine, a little over three quarters of respondents replied in the affirmative. However, vaccination costs, current export requirements for a negative Hendra virus antibody status, and perceived safety issues especially in pregnant mares threaten to limit uptake. Because Hendra virus is not highly infectious, the concept of ‘herd immunity’ does not apply. Every individual horse is potentially at risk of infection from the natural flying-fox reservoir, and because vaccination efficacy can never be regarded as 100%, minimising exposure risk should remain a fundamental component of the property-level risk management strategy.

## Conclusion

We chose to focus our analysis on knowledge uptake, risk perception, and risk mitigation. We found inconsistent awareness and/or adoption of available knowledge by horse owners, suggesting the need for review of current communication strategies. We also found enduring confusion in relation to Hendra virus risk perception, with both over-and under-estimation of true risk. Finally, we found considerable lag in the uptake of recommended risk minimisation strategies by horse owners, notwithstanding their apparent ready implementation. In contrast to this however, we identified frustration and potential alienation by horse owners who found the recommended strategies not feasible. While the focus of this study has been Hendra virus and horse owners, the insights gained could readily apply to other complex risk-management scenarios. Fundamentally, sound knowledge underpins valid risk perception, which in turn forms the basis of effective risk management strategy. Sound knowledge reflects effective communication, while attitudes and opinions reflect, at least in part, knowledge synthesis in the context of individual and social circumstances and values. This survey was the first of its kind in terms of scope and scale, and it has provided a unique insight into the human component of Hendra virus risk management.
